# Cellular Mechanisms of Ciliary Length Control

**DOI:** 10.3390/cells5010006

**Published:** 2016-01-29

**Authors:** Jacob Keeling, Leonidas Tsiokas, Dipak Maskey

**Affiliations:** Department of Cell Biology, University of Oklahoma Health Sciences Center, 975 NE 10th Street, Oklahoma City, OK 73104, USA; jacob-keeling@ouhsc.edu (J.k.); ltsiokas@ouhsc.edu (L.T.)

**Keywords:** Ciliogenesis, ciliary vesicles, IFT, cell cycle, centrioles, basal body

## Abstract

Cilia and flagella are evolutionarily conserved, membrane-bound, microtubule-based organelles on the surface of most eukaryotic cells. They play important roles in coordinating a variety of signaling pathways during growth, development, cell mobility, and tissue homeostasis. Defects in ciliary structure or function are associated with multiple human disorders called ciliopathies. These diseases affect diverse tissues, including, but not limited to the eyes, kidneys, brain, and lungs. Many processes must be coordinated simultaneously in order to initiate ciliogenesis. These include cell cycle, vesicular trafficking, and axonemal extension. Centrioles play a central role in both cell cycle progression and ciliogenesis, making the transition between basal bodies and mitotic spindle organizers integral to both processes. The maturation of centrioles involves a functional shift from cell division toward cilium nucleation which takes place concurrently with its migration and fusion to the plasma membrane. Several proteinaceous structures of the distal appendages in mother centrioles are required for this docking process. Ciliary assembly and maintenance requires a precise balance between two indispensable processes; so called assembly and disassembly. The interplay between them determines the length of the resulting cilia. These processes require a highly conserved transport system to provide the necessary substances at the tips of the cilia and to recycle ciliary turnover products to the base using a based microtubule intraflagellar transport (IFT) system. In this review; we discuss the stages of ciliogenesis as well as mechanisms controlling the lengths of assembled cilia.

## 1. Introduction

Cilia are dynamic microtubule (MT)-based organelles that emanate from the surface of many eukaryotic cells, ranging from the green algae *Chlamydomanas reinhardtii* to most quiescent, differentiated cells in the human body [1,2]. As the primary cilium has been recently shown to be critical for multiple metazoan processes such as organ development, cell differentiation, and cell polarity [3,4]; it is interesting to consider that while most cells have the capacity to form cilia [5], not all cells retain primary cilia at all times. Defects in primary cilium assembly have been associated with common genetic disorders such as human cystic kidney disease, obesity, mental retardation, blindness as well as various other developmental malformations [3,6]. In general, these human disorders are classified as ciliopathies. In addition, genetic studies in mice have demonstrated that cilia are essential for the function of the hedgehog (Hh) and wnt pathways, and contribute to the organization of the body plan, as well as tumorigenesis [7,8]. Conversely, most (although not all) cancer cells lack cilia [9]. Therefore, there has been great interest in identifying factors that regulate not just ciliary assembly and disassembly, but also ciliary length, which provides the physical scaffold for a cilia-associated signaling system [10,11,12]. In this review, we focus primarily on recent advances in our understanding of the stages of ciliogenesis and on ciliary length control mechanisms.

## 2. The Cilium: Types and Structure

**Types**: Cilia are broadly divided into two types: motile and primary; both types function as sensory organelles that register alterations in the extracellular milieu and relay information into the cell to control processes in development and tissue homeostasis [13,14,15]. Most motile cilia are built with nine doublet microtubules surrounding a central pair of singlet microtubules (9+2). In some cell types motile cilia can appear as multi-ciliated bundles, such as in the respiratory epithelium. Dynein arms anchored to the outer axoneme of these motile cilia can cause a synchronized sliding of the axonemal microtubules to generate a coordinated beating motion in the same direction as their neighbors which serves to generate directed physical flow such as is utilized for moving mucus in the respiratory tract or cerebrospinal fluid in the central nervous system [13]. The nodal cilium responsible for establishing left-right asymmetry within the developing embryo is a unique type of motile cilium. These cilia beat in a rotational motion and although this movement is still generated by axonemal dyneins, nodal cilia lack the central pair of microtubules and exist as a (9+0) cilia [16]. The axoneme of a primary cilium is also composed of only nine outer sets of microtubules as the (9+0) axoneme; however, this cilium lacks the anchored dynein that is responsible for the directional movement seen in its motile cousins [13]. The primary cilium is solitary and non-motile. It can be found in almost all other mammalian cell types and has essential functions in multiple signaling pathways [3,11,13].

**Structure:** The core of the cilium consists of the microtubular axoneme, and the origin of this core structure is a modified centriole, which forms the base of the cilium [17,18,19]. In keeping with its location, the name for this organelle once centriolar differentiation is complete is the basal body. During cell division, the centrosome serves as a microtubule-organizing center or spindle pole body [20,21]. Each centrosome consists of two centrioles embedded in a peri-centriolar matrix (PCM). The older of the two centrioles is referred to as the mature or mother centriole, which carries distal and sub-distal appendages. The younger centriole is referred to as the daughter centriole, and the two centrioles can be distinguished from each other by staining for centrosomal marker proteins [22]. As cells exit from the cell cycle, the centrosome differentiates into a basal body to initiate the cilia formation [17].

Reversible post-translational modification of tubulin protein subunits helps produce functional ciliary microtubules and effects the biochemical properties of the axoneme [23]. The various post-translational modifications including: acetylation, palmitoylation, tyrosination/detyrosination, glutamylation, and glycylation help to co-regulate ciliary stability and motility [23,24,25]. The acetylation of microtubules is the most frequent post-translational modification associated with microtubule stabilization [26]. However, it is believed that that this modification does not directly increase stability [25,27]. It has been thought that detyrosination may stabilize the axonemal fiber by removing the tyrosine residue at the C-terminus of tubulin subunits. On the other hand, other modifications such as polyglutamylation and polyglycylation could modulate the recruitment process of proteins to the axoneme, causing indirect changes in the ciliary structure [28].

## 3. Cilia and the Cell Cycle

The formation of the primary cilium is inversely correlated with cell cycle progression. Typically, initiation occurs after a cell has completed mitosis and enters the G0/G1 phase of the cell cycle. Cilia become shorter rapidly as cells progress from G1 to S and are practically invisible in mitosis [29,30]. It has been proposed that cilia are a negative regulator of the cell cycle because the ciliary basal body competes with mitotic machinery for the use of centrioles. In particular, it is thought that cilia themselves can influence specific stages of the cell cycle, such as the G1 to S transition [17] or the M to G1 transition [31]. Here, we focus on the G1 to S transition. Given the indispensable and transient nature of the organelle, it is not surprising that ciliary assembly and disassembly is precisely coordinated with cell cycle progression. The mechanisms that trigger the cells to enter into the G0/G1 phase probably are intimately linked to the initiation of ciliogenesis. Cilia are not compatible with mitotic spindle formation with the exception of some unicellular organisms and in insect (namely butterfly) spermatogenesis. Thus, cilia must typically be disassembled as cells re-enter the cell cycle [17,32]. Therefore, it is important to know how ciliary assembly and resorption is able to influence cell cycle re-entry.

**Figure 1:**
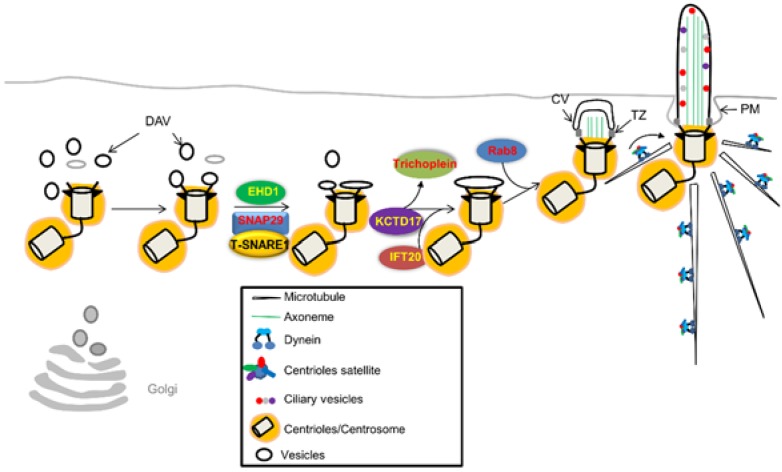
A schematic of basal body docking and ciliogenesis. As the mother centriole begins its differentiation and migration to the cell surface it acquires distal appendenges and distal appendage vesicles (DAV) from the golgi. Proteins such as EHD1 in conjunction with Rab8 help to expand the DAV into a ciliary vesicle (CV). As ciliogenesis continues the basal body acquires positive regulators of ciliogenesis and removes negative regulators such as Trichoplein, allowing docking and the formation of the transition zone (TZ). Once the basal body docks at the plasma membrane (PM) axonemal extension can take place into the extra-cellular space.

Initiation of ciliogenesis is a well-orchestrated process where Golgi-derived ciliary vesicles attach to the distal ends of the mother centriole (Figure 1). This can occur either by vesicle fusion with the basal body en route to the cell surface or by direct contact and fusion of the basal body to the plasma membrane [33]. If a primary cilium is to be formed, the mother centriole must first differentiate into a basal body. This process is associated with the gain of non-centriolar structures such as the basal foot and the transition fibers needed to anchor the centriole in place and to regulate the contents of the completed cilium after a stable length has been achieved [18,34]. After the mother centriole has become a fully differentiated basal body, ciliogenesis is then initiated by the migration and docking of the basal body along with its vesicles onto the plasma membrane [19].

Upon cell-cycle re-entry, the balance of cilium assembly and disassembly is shifted towards disassembly and ciliary resorption begins [30,35]. Most of ciliary resorption studies have been conducted in cell culture, where cells were synchronized at G0/G1 by serum starvation and then forced to re-enter the cell cycle using serum or growth factors. At the end of resorption, the basal body is released from the plasma membrane where it once docked allowing the centrioles to once again function as microtubule organizing center (MTOC) during mitosis [18,22].

## 4. Programs of Ciliogenesis

The formation of primary cilia can occur through two distinct pathways, the so-called extracellular and intracellular pathways, depending on the cell [36,37]. As an example of the extracellular pathway, in epithelial cells of the kidney or lung, the basal body fuses with the apical surface of the plasma membrane. From this point the cilium protrudes directly into the extracellular space as it elongates [19,38,39]. Conversely, in the intracellular pathway, which can be found in fibroblasts and neuronal precursor cells, the basal body associates with a cytoplasmic vesicle *en route* to the plasma membrane. The cilium begins to grow from this initial vesicle while additional vesicles supply membrane to support the growing axoneme until the vesicular structure comes in contact with and fuses with the plasma membrane (Figure 1) [36,37,40]. This mechanism of ciliogenesis often results in a ciliary pocket, or ciliary pit in which a portion of the mature cilium rests within a recessed area that would typically be cytoplasmic in a non-ciliated cell. In these instances the basal body is positioned deeper within the cells than is seen in the extracellular mechanism of ciliogenesis [36,37].

Other conditions that have been shown to induce the formation of the primary cilium are starvation by growth factor depleted media and cell confluency [41,42], both classically thought to act by forcing the cell into a non-mitotic state. However, some studies have shown that the ciliation due to confluency is also partially due to changes in the actin cytoskeleton. Moreover, it has been shown that when subjected to shear stress, endothelial cells will resorb their cilia [43]. To add further insight to this finding, Pivatal *et al.* recently demonstratedthat cell spreading and contractile state have a strong influence on the formation of the primary cilium [44]. Pivatal *et al.* have shown that cells that are highly spread, thus forming a high density of contractile bundles of F-actin are much less likely to form a primary cilium. In contrast to this finding, Boisvieux-Ulrich *et al.* have shown that in quail oviduct organ cultures, treatment with the same F-actin inhibitor used in the Pivatal *et al.* (2010) study (Cytochalasin D) inhibited ciliogenesis by preventing docking of the basal body with the plasma membrane [45]. Further study is needed to clarify this discrepancy and the role that the actin cytoskeleton plays in ciliogenesis. However, this connection between contractile state and cell spreading is especially interesting when one considers that the Hippo pathway, which is closely correlated with growth and organ size, is activated in the context of a highly spread, highly adherent cell [46,47]. Indeed, the ciliary protein NPHP4 which is mutated in patients with the kidney disease nephronophthisis is a powerful inhibitor of the Hippo pathway; this protein has also been shown to interact with planar cell polarity proteins aid in the organization of the subapical actin network [48,49]. It will be interesting to see if other cues for ciliogenesis exist and to further investigate the crosstalk between cilia formation and Hippo pathway activation.

### 4.1. Initiation of Ciliary Assembly

The distal appendages or transition fibers of the mature basal body have been implicated in the attachment process although the complete mechanism of attachment has yet to be elucidated [50,51]. Nigg and colleagues found the centrosomal protein Cep164, a marker for distal appendages, is a major structural component for cilia formation [50,52]. In addition, Pereira and colleagues demonstrated that Cep164 is an indispensable component for the docking of the vesicles at the mother centriole. Cep164 helps to promote the association of ciliary vesicles to the distal appendages by interacting with a small GTPase, Rab8a and its guanine nucleotide exchange factor (GEF), Rabin8 [52]. These molecules are essential for the vesicular trafficking needed to build a ciliary membrane [36,37]. Large ciliary vesicles (CV) accumulate in this region and fuse to the newly formed membrane around the elongating axoneme thereby creating a sheath around the cilia [33,53]. This axoneme elongating process continues until the membrane-bound axoneme reaches the cell surface and fuses with the plasma membrane, allowing the cilium to be exposed to the extra-cellular milieu [33,36]. Cep164-depleted cells accelerate the cell cycle but inhibit overall proliferation. This apparent paradox is due to Cep164 function also being associated with DNA damage-induced replicative stress, apoptosis, and epithelial-to-mesenchymal transition, which could contribute to the pathological mechanisms of the polycystic kidney disease (PKD) or nephronophthisis [54].

In addition to Cep164, several other transition fiber/distal appendage proteins have been identified, including Cep89 (CCDC123), Cep83 (CCDC41), SCLT1, OFD1, OFD2, and FBF1/Albatross. An elegant study from Tanos *et al.* has revealed an essential hierarchy of distal appendage assembly. Cep83 is required to recruit both Cep89 and SCLT1. SCLT1 is then needed to bring in Cep164 and FBF1 [55]. This group has also shown that CEP83 is required for the docking process and that its downstream binding partners, while not needed for docking per se, are required for the removal of ciliogenesis inhibitor CP110 and subsequent axoneme extension [55]. Talpid3 is another centrosomal protein implicated in mediating the interaction between distal centriole appendages and vesicles [56,57]. Depletion of this protein is sufficient to arrest development of the primary cilium, but there is no clear evidence that Talpid3 promotes or mediates the docking of vesicles to the distal end of the basal body; thus, its function is most likely in some other facet of ciliogenesis [56].

After distal appendage formation has been completed, these complexes interact with a post-Golgi vesicle on the mother centriole to form a ciliary vesicle. It has been reported that the small GTPase, Rab8a and its GEF, Rabin8, have an important role in ciliary vesicle formation and extension [58]. GTP-bound Rab11 interacts with Rab8 to regulate vesicle transport from the *trans*-Golgi network and recycling endosomes during ciliary assembly [10,58,59]. In addition, Rab8 interacts with the TRAPPII to form complexes that regulate intra-Golgi transport through vesicle tethering [59]. Ahi1 (or Jouberin), which is mutated in Joubert syndrome, regulates the recruitment of Rab8a to the basal body [60,61]. In exocytosis, sequential activation of the small G-proteins, Rab11 and Rab8a, has been well documented [62]. Once activated this complex recruits Sec15 and myosin, a component of the exocyst vesicle and the actin motor protein, respectively. These facilitate tethering and transport during early stages of cilia assembly [62,63,64]. During the developmental stage of outer photoreceptor cells, it is believed that Rab8 conjugates with the specific SNARE proteins, syntaxin 3 and SNAP-25 to promote expansion of the ciliary membrane by vesicular fusion [65]. Recently, membrane shaping proteins EHD1 and EHD3, in association with the Rab-11-Rab8 cascade, have been found to be required for early vesicle-mediated ciliary assembly at the distal appendages [53]. Finally, several accessary structures are formed subsequent to basal body differentiation and docking, such as the transition zone, rootlets and basal feet that provide structural support to the cilium [66]. These data suggest that centriole-to-membrane docking mediated by distal appendage proteins may serve as an instructive signal that temporally and spatially regulates cilia initiation.

### 4.2. Maintenance of Ciliary Length

Once the basal body has been correctly positioned on the apical surface of the plasma membrane, axonemal extension can take place. Axonemal extension occurs exclusively at the plus ends of the microtubules, with vesicles derived from the Golgi apparatus serving to increase the membrane content of the organelle concurrently with extension [19,33]. This presents an initial obstacle and opportunity for regulation since protein synthesis occurs within the cytoplasm while access via diffusion has been substantially limited by the basal body docking and subsequent formation of the ciliary transition zone [67]. Thus, axoneme extension relies on the Intraflagellar Transport (IFT) system with the malarial pathogen *Plasmodium falciparum* serving as one of the only known, although notable, exceptions to this rule [68]. *Drosophila melanogaster* sperm flagella also seem to be IFT independent. The knock down of the essential ciliary IFT gene IFT88/Polaris results in a loss of cilia on fly sensory cells but no significant change of morphology or function of sperm flagellum [69]. Although the unique properties of each organism and organelle give intriguing hints as to why these structures may require alternate mechanisms of assembly, they are not be discussed further in this review.

The IFT system is an evolutionarily conserved system that is specialized for transport of proteins into and out of the cilium. Our current understanding of the IFT system is based on extensive experiments carried out in the algae *Chlamydomonas reinhardtii* and subsequently in *C. elegans* [70,71,72]. The IFT system consists of two multi-protein complexes, IFT-A and IFT-B, which are made up of approximately two dozen known members in total that travel back and forth within the cilia. These complexes are proposed to form at the basal body before entry into through the transition zone into the cilium proper, and Pericentrin has been shown to be required for the localization of IFT proteins at the cilia base [73,74]. Although these complexes are thought to travel together, different members of each complex have been proposed to serve broadly distinct roles. IFT-B is classically associated with anterograde transport of ciliary precursors as demonstrated by a number of studies where mutants of IFT-B were shown to have defective or eliminated ciliogenesis [75]. IFT-A has been shown to be involved in the retrograde transport and recycling of ciliary components. One of the earliest experiments demonstrating retrograde transport in IFT-A was done via a temperature sensitive assay in *Chlamydomonas* where some IFT-A knock outs result in a broadly similar constellation of ciliary defects of bulged membranes combined with defective retrograde transport velocities and frequencies [76]. However, in support of the complexes traveling in tandem, it has been shown that Kinesin-2, the classic anterograde motor, physically binds to and carries complex A to the tip of the cilium. This was found by disruption of the BBSome in *C. elegans* which causes a species specific separation of the two complexes within the cilia [77]. There are, as always, notable exceptions to this rule with some membrane proteins such as Arl13b and Smo showing reduced ciliary transport in the absence of the IFT-A protein IFT144 [78]. Also the IFT-B proteins IFT25 and IFT27 have had demonstrated roles in the ciliary export of the hedgehog components Smo and Ptch1 [79,80]. However as both the complexes do recycle up and down the cilium together it is certainly not impossible for proteins in either complex to function in both facets of transport, thus it may be more proper to refer to anterograde and retrograde complexes rather than A or B subunits.

The cargoes and binding sites of these complexes are currently being investigated, and protein-protein interaction experiments are aided by screens for proteins that exhibit IFT-like transport velocities through the cilium. For instance, as it pertains to ciliary length, tubulin has been shown to have direct binding affinity to IFT81 and IFT74 [81]. Although there are several ciliary proteins that can be screened in this way, there are also numerous proteins, including tubulin, that appear to move into and through the cilium by diffusion and it has been shown that cargo from the anterograde complex is progressively unloaded and re-loaded throughout the journey up the axoneme [67,82,83]. These alternatives add interesting wrinkles and make the matter of ciliary protein transport more complex to study.

The active anterograde complex is carried to the ciliary tip by the plus end directed motor Kinesin 2. Kinesin 2 exists in two major forms, heterotrimeric and homodimeric Kinesin 2. Heterotrimeric Kinesin 2 forms a complex with two motor subunits and an accessory protein known fittingly as Kinesin Associated Protein 3 (KAP3) [84]. Heterotrimeric Kinesin 2 is necessary for ciliogenesis in most organisms with the exception of *C. elegans* where the function of homodimeric Kinesin 2, known as OSM-3 in worms, is sufficient to allow ciliation [85]. Thus there may be some observations made in the nematode that may not necessarily apply to other eukaryotic systems due to *C. elegans* specific functions or redundancies. Once the complexes have reached the tip of the cilium, the anterograde complex presumably becomes inactive, unloads its cargo and subsequently the retrograde complex becomes active and carries the complexes back to the basal body by Dynein 2 along with any cargo that is to be exported from the cilium [86].

In experiments where the primary cilium is allowed to develop normally and then members of the anterograde IFT are knocked down, cilia have been observed to progressively shorten. Interestingly enough, it seems that the constant rate of cilia disassembly may not be spontaneous, instead relying on the activity of another kinesin. In the protozoa *Leishmania major*, Kinesin 13, which has a recognized microtubule depolymerizing ability, was shown to localize to the tip of the flagellum during ciliation and overexpression or knock-down of the protein produced shortened or lengthened cilia, respectively [87]. Kif24 in RPE cells, a kinesin-13 family protein with microtubule-depolymerizing activities, seems to work in a similar fashion [88]. This idea of an active depolymerizing agent is strengthened by data showing that sperm flagella lack typical IFT for maintenance and yet retain their steady state axoneme much longer temporally than is seen in IFT knock downs of the primary cilium [89]. However, it is important to remain aware that sperm may also be subject to additional stabilization mechanisms that may be incompatible with diploid cells that may, at some point, choose to re-enter the cell cycle.

### 4.3. Regulators of Ciliogenesis

A set of robust regulatory control mechanisms is required in order to suppress the untimely conversion of the mother centrioles into basal bodies in dividing cells. This effect can be achieved through recruitment of positive regulators and destruction of negative regulators of ciliogenesis in earlier stages of basal body conversion (Figure 1). Positive regulators of ciliogenesis should remain at high levels throughout ciliogenesis and negative regulators of ciliogenesis should remain low or be eliminated during the stages of ciliogenesis and ciliary maintenance [18,35]. Research into this area has revealed several key proteins as these regulators.

#### 4.3.1. CP110 Destruction/Dislocation by TTBK2 and MARK4

CP110 is localized to the distal ends of centrioles, forming a “cap” above the growing microtubules that inhibits microtubule growth [90], suggesting that CP110 acts as a negative regulator of cilia formation, and its abundance is associated with decreased cilia assembly by blocking centriole elongation [91,92]. Kobayayashi *et al.* have shown that Kif24 interacts with CP110 and Cep97 and localizes to mother centrioles. Depletion of Kif24 in cycling cells leads to disappearance of CP110 from mother centrioles and subsequently to aberrant cilia formation. However, this did not promote the growth of abnormally long centrioles, unlike depletion of CP110 and Cep97 [88] suggesting that Kif24 specifically remodels centriolar microtubules without significantly altering cytoplasmic microtubules. CP110 also associates with Cep290 and Cep104 [93,94]. It has been shown that CP110 restrains Cep290 from promoting the early stages of ciliogenesis in proliferating cells. However, once a cell exits from the cell cycle, the loss of the CP110 protein releases Cep290 from inhibition [92]. It has been shown that CP110 stability is regulated by Cep97. Before ciliation, CP110 and Cep97 must be eliminated from the maternal centrioles [91]. It has been demonstrated that two kinases, Tau tubulin kinase 2 (TTBK2) and microtubule-associated protein/microtubule affinity regulating kinase 4 (MARK4) are involved in the initiation of ciliogenesis by excluding CP110 from mother centrioles [95]. Anderson's group demonstrated that TTBK2, a spinocerebellar ataxia-associated protein, acts at the distal ends of the basal body, promoting removal of CP110 and recruitment of IFT proteins, which then build the ciliary axoneme [96]. In response to cell-cycle exit, TTBK2 is recruited to the mother centriole preceding the removal of CP110 to initiate the ciliogenesis. TTBK2 may phosphorylate one or more proteins of the CP110/Cep97/Cep290/Kif24 cilia-suppression pathway. Furthermore, MARK4, a Ser/Thr kinase, acts as positive regulator of ciliary assembly [88,92,95]. MARK4-depleted cells impair the elimination of the CP110-Cep97 complex from basal bodies, and consequently, elongation of the axoneme fails to occur [95]. However, basal body attachment to ciliary vesicles was not impaired in MARK4-depleted cells [95,97]. The precise molecular mechanisms of the removal of CP110 by TTBK2 and MARK4 remain still unclear.

#### 4.3.2. Trichoplein is Degraded by CRL3^KCTD17^

Besides CP110, several additional proteins are likely negative regulators of ciliogenesis. For example, Trichoplein, which is localized at sub-distal/medial zone of both mother and daughter centrioles and activates centriolar Aurora-A kinase in the cycling cell [98]. Trichoplein disappears from mother centrioles during ciliogenesis, whereas overexpression blocks ciliogenesis, suggesting that trichoplein is a negative regulator of this process [98]. Degradation of trichoplein takes place through the action of the Ubiquitin-proteasome system (UPS) at an early stage of ciliogenesis via the activity of E3 complex CRL3-KCTD17 [99].

#### 4.3.3. Ofd1 is Removed by Autophagy

It has been shown that Oral-facial-digital syndrome 1 (OFD1), acts at the distal centrioles to build distal appendages, recruit IFT88, stabilize centriolar microtubules, and is required for primary cilia formation [100,101]. Ofd1 localizes to centriolar satellites, interacting with PCM1, Cep290, and Bardet-Biedl syndrome 4 (BBS4) [102]. Ofd1 is involved in centriole length control by recruitment of BBS4 to cilia [100]. Ofd1 is removed from centriolar satellites through selective autophagy that can be induced during serum starvation. As a result, longer cilia are formed under this condition [103]. Therefore, Ofd1 at centriolar satellites has a crucial role for the suppression of ciliogenesis whereas Ofd1 at centrioles is necessary for ciliogenesis [103].

The proteins which are involved in recruitment and formation of basal body appendages are required for ciliogenesis. For example, Outer dense fiber protein 2 (ODF2) has been shown to be required for distal and sub-distal appendages and for ciliogenesis in mouse cells [97]. However, the mechanistic roles for these proteins are not known. These data suggest that the proper formation of distal ends and correct regulation of centriolar length are important for efficient cilia assembly [18]. These mechanisms seem to impact different stages of ciliogenesis in a complementary fashion allowing for proper cilium biogenesis and function.

Despite these developments, important molecular mechanisms involved in the initiation of cilia formation still remain unknown. For example, it is not known how distal appendages are anchored to ciliary vesicles or the plasma membrane and how positive and negative regulators are recruited and eliminated at the onset of basal body formation at earlier stages of ciliogenesis. More work into this area should help shed some light on these mysteries.

## 5. Ciliary Length Control Mechanisms

Given the fact that if anterograde IFT is inhibited cilia resorb at a constant rate [104], subunits coming off must always match subunits being added, thus achieving a state of “dynamic stability” [104,105]. There are multiple steps where this regulation could occur by mechanisms that are incompletely understood. This control could take place at the transcriptional level. Although this has not been strictly related to steady state length control, increased transcription of the dynein 2 subunit DYNC2H1 is associated with ciliary disassembly in sea urchin [106]. Another step where regulation could occur is the transport and entry of IFT complexes and precursors into the cilium. In ciliated cells, it has been proposed that IFT molecules are the main regulators of ciliary growth. In the flagella of C*hlamydomonas* it has been shown that accumulation or increased activity of the anterograde IFT complex leads to further elongation of the cilia, whereas a decrease in the mobility of the these complexes leads to the generation of shorter cilia [105,107]. A similar observation has been recapitulated in mammalian cells, where increasing the mobility of the anterograde IFT complex, as measured by IFT88 velocities, generated longer cilia [108]. In this study cilia were found to lengthen with either calcium release inhibition or chemical stimulation of cAMP. These changes in length are believed to be upstream of PKA activation. It has been shown that ablation of Tctex-1, a putative component of the IFT-associated dynein, also produces longer cilia [109]. Finally, over-production of soluble tubulin, the building block of axoneme, leads to increased ciliary length, whereas limiting the supply of tubulin by treating the cells with microtubule stabilizing drug taxol leads to shortened cilia or no cilia at all [110,111].

The BBSome has been commonly implicated in ciliogenesis, with defects in BBSome components resulting in IFT and morphological defects of the cilium of *C. Elegans.* In these animals, disruption of bbs-1 bbs-7 and bbs-8 causes a separation of the A and B complexes with Kinesin-2 remaining attached to IFT-A while IFT-B is carried upwards by OSM-3 [85,112,113]. In other systems such as the mouse, the structural defects seem to be much more milder or absent in primary cilia. However, a lengthened and bulged appearance in motile ependymal cilia and defects in sperm flagellar assembly have been reported. Apparent defects in the gating of some membrane proteins such as SSTR3 and MCHR1, and defective export of others such as Ptch1 and Smo are more consistent in primary cilia BBSome mutants. Thus this seems to be a more likely mechanism of action by which the BSSome acts within primary cilia of other eukaryotes [80,114,115,116,117]. Phosphorylation patterns produced on Kinesin 2 by various kinases have been shown to affect Kinesin motor activity and thus anterograde IFT activity. The MAP kinases and GSK-3β have been shown to have regulatory roles in ciliary length [118,119,120]. Post-translational modifications of tubulin in the axoneme are also potential candidates for length regulation. Polyglutamylation and detyrosination have been shown to affect the motor function of Kinesin 2 [121,122]. Kif7 has recently been reported to localize to the ciliary tip and to modulate hedgehog signaling at least partially by inducing tubulin depolymerization [12]. When one includes these numerous observations with the aforementioned flagellar tip disassembly protein, Kinesin 13 [87], it seems likely that the cell can exercise much better control over the final length of the cilia than by simply letting monomer concentrations determine the polymer kinetics of such an important structure. The fact that different cell types within an organism and between different species have varying average primary cilia lengths gives credence to the idea of a vast multi-factorial regulation process that is active in one degree or another in different contexts throughout different cells. One major question still open to the field and relevant to this discussion is what overarching environmental and cellular cues might cause the cell to extend or contract these antennae.

### 5.1. Nde1 is Regulated by CDK5-SCF^fbw7^

It has been demonstrated that the mother centrosomal protein Nde1, Nuclear Distribution gene E homologue 1, is a negative regulator of ciliary length [123]. Cells depleted of Nde1 have longer cilia and exhibit a delay in cell cycle re-entry that correlates with increased ciliary length. Overexpression of Nde1 shortens the ciliary length through its association with the dynein light chain protein, DYNLL1/LC8. In addition, simultaneous depletion of IFT88 or IFT20 suppresses cilia formation and reverses the effect of Nde1 depletion on the rate of cell cycle re-entry suggesting that Nde1 affects cell cycle re-entry through cilia [123]. Maskey *et al.* have identified F-box and WD40 repeat domain-containing 7 (Fbwx7) as a E3 ubiquitin ligase that mediates the destruction of Nde1 and maintains low levels of Nde1 at G1/G0 phase of cell cycle, allowing these cells to form proper and fully functional cilia. Maskey *et al.* have demonstrated that NDE1 is phosphorylated by CDK5, a kinase active in G1/G0, priming NDE1 for recognition by the FBW7 E3 ubiquitin ligase, and subsequently targeting it for degradation by the Ubiquitin-proteasome system [124]. SCF^Fbw7^ is a tumor suppressor protein which targets several proteins such as c-Myc, Notch1, c-Jun and cyclin E, for destruction and is involved in the maintenance of normal stem cells and cancer initiating cells [125,126]. These data suggest that the CDK5-SCF^Fbw7^-Nde1 pathway is not only important for the regulation of ciliary length by cell cycle but also critical for crosstalk between cilia and the cell cycle in normal and cancer stem cells.

### 5.2. APC in Ciliogenesis

**Figure 2:**
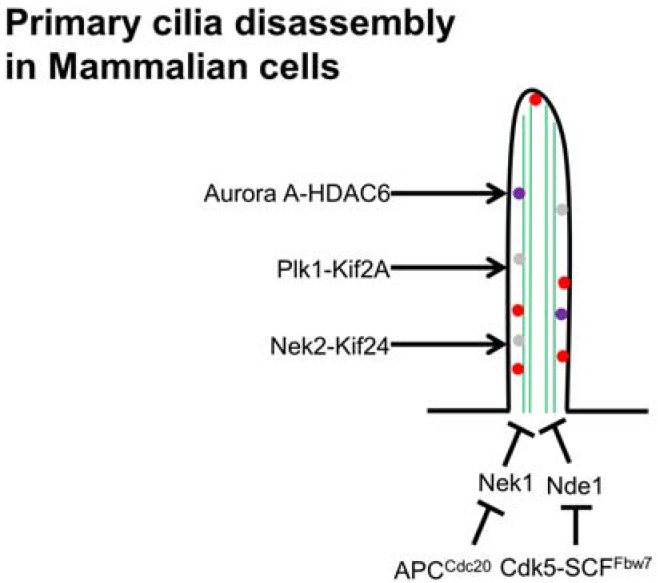
An illustration of molecular mechanisms of primary cilia disassembly (along the side) in mammalian cells and possible ciliary length control mechanisms (lower portion).

The Anaphase Promoting Complex (APC), a key E3 ubiquitin ligase required at the onset of anaphase, localizes to the basal body and has an important role in regulating ciliary polarity [127]. Kirschner and co-workers found that after serum stimulation, the activity of APC^cdc20^ is required for proper maintenance of ciliary length as well as for the timely resorption of cilium [128]. They have demonstrated that APC^cdc20^ regulates the stability of axonemal microtubules through targeting Nek1 (Figure 2) for ubiquitin-mediated proteolysis [128]. In contrast, BUBR1-dependent Cdc20 degradation in G0 phase cells plays an important role in the maintenance of APC^CDH1^ activity which promotes the assembly of primary cilia [129]. However, how these activities occur needs to be investigated.

### 5.3. Arf and Arl Members in Length Control

Several members of the Arf (ADP-ribosylation factor)/Arl (Arf-like)- family small GTPases, including Arf4, Arl3, Arl6 and Arl13b have cilium-associated functions [130,131,132]. Arf4 acts via trafficking to the cilliary base, Arl3 and Arl13b are ciliary proteins with links to IFT [116,133,134]. Arl13b localizes in cilia and regulates ciliary length. It has been shown that loss of Arl13b led to shortened cilia and reduced number of cilia *in vitro*, whereas overexpression of Arl13b caused increased ciliary length suggesting that Arl13b has an important role in ciliary length control [135]. The authors have proposed that Arl13b works in a similar manner to the GTPase Arl6 which recruits trafficking proteins of the Bardet-Biedl syndrome complex to the cilium and regulates ciliary length [116,136]. The mammalian orthologue of Arl13b in *C. elegans*, Arl13, seems to stabilize IFT [137,138]. In the work by Cevik *et al.* [138], an in frame deletion of amino acids 169-342 of this protein showed delayed dye filling, disrupted cilia structure, and abnormal ciliary accumulation the PKD2 protein. However, further work by Larkins *et al.* show that the mammalian version of the protein, when knocked down, did not seem to affect IFT via IFT88 imaging [135]. However, these data are particularly difficult to interpret as the truncated worm protein may have attained some unexpected dominant negative effects and in the knock down assay some residual Arl31b remained in the cells. Extrapolation from worm to mammalian systems is made even more difficult due to the fundamental differences in the IFT trains. This being said, mammalian cells do show an abnormal accumulation of the hedgehog activator Smo. So there does seem to be a consistent effect at least in the gating of proteins into or out of the cilium.

## 6. Cilia Disassembly

When a ciliated cell commits to re-entering the cell cycle or undergoes some form of stress such as sheer stress, cilia must be resorbed so the cell can separate and relocate the mother and daughter centrioles to opposite poles of the cell cortex for mitosis. Before the basal bodies can uproot themselves from the apical surface of the cell, the cilium must first be resorbed [21,139]. Most of ciliary resorption studies have been conducted in cell culture, where cells were synchronized at G0/G1 by serum starvation and then allowed to synchronously re-enter the cell cycle using serum or growth factors [30]. It has been shown that growth factor stimulation under these conditions triggers sequential activation of several proteins involved with cilia resorption, including Human Enhancer of Filamentation 1 (HEF1), Aurora A kinase, Pitchfork (Pifo), and Tctex-1 [140,141,142]. Pugacheva *et al.* have shown that growth factor stimulation of serum-deprived cells induces activation of HEF1 and Aurora A kinase. Thereafter, Aurora A activates histone deacetylase 6 (HDAC6), resulting in deacetylation of axonemal microtubules, facilitating ciliary resorption. Inhibition of Aurora A kinase or HEF1 blocks cilia disassembly, whereas this process is accelerated when constitutively active variants of these proteins are expressed [140]. In addition to HEF1, Pitchfork (Pifo), which is specifically expressed at the basal body in embryonic node, interacts with Aurora A and facilitates cilia disassembly [141]. Moreover, Polo-like kinase 1 (Plk1) may directly phosphorylate HDAC6, stabilizing HEF1 and facilitating cilia disassembly [143]. In *Chlamydomonas*, phosphorylation of aurora-like kinase (CALK) is used as a marker of flagellar length in both assembly and disassembly process [144]. (Figure 2) Tctex-1, when phosphorylated at T94, is shown to be enriched in the transition zones during ciliary resorption and disassembly. Phospho-mimetics of this protein are shown to increase the proportion of actively cycling cells and knock down of this protein causes a decrease in cellular division that can be rescued via artificial knock down of the primary cilium, thus strengthening the observation that Tctex-1 mediates an effect on the cell cycle through the cilium [142].

Besides *pifo*, a mutation in Inositol polyphosphate-5-phosphatase E (INPP5E) has been reported in patients with Joubert syndrome. INPP5E is localized at the cilia, and is involved in ciliary disassembly [145,146]. Inactivation of *INPP5E* in mice shows multiorgan disorders associated with structural defects of the primary cilium. However, the percentage of ciliated cells and ciliary length of ciliated cells are not affected in INPP5E- depleted cells suggesting that the INPP5E levels in the ciliary pool maintain an appropriate balance of phospholipids. However, mutations in this protein show that any disturbances of this lipid balance could potentially lead to acceleration of ciliary resorption and cell cycle re-entry in response to mitogenic stimuli [145,146]. More work is needed to better characterize and differentiate the roles of this protein in cilia maintenance and signaling.

It was observed that following cell division, *C. reinhardtii* leaves flagellar fragments lodged in the cell wall. This led to the question of whether ciliary resorption is actually the sole cause of basal body disassociation from the plasma membrane during mitosis. The protein responsible for this severing was shown to be katanin and it has been shown in algae*,* that katanin can be used to sever the doublet microtubules emanating from the basal body thus freeing the organelle for withdrawal into the cytoplasm where it can participate in cell division [147,148]. Katanin was first discovered in 1993 in Xenopus as a novel taxol insensitive ATPase dependent microtubule severing protein [149]. Since then katanin has been shown to play a role in the cilium as well as in the more typical cytoskeleton. Sudo *et al.* show in their studies that katanin functions in neurons and fibroblasts and preferentially severs acetylated microtubules [150]. Thus, in algae, at least, katanin seems to be a critical component involved in the final step of cilia disassembly via facilitation of basal body disassociation. This mechanism seems to at least be partially retained through phylogeny as in the mouse it has been shown that the knock down of katanin p80 results in aberrant centriole duplication and supernumery cilia in association with holoprosencephaly and defects in hedgehog signaling [151]. This finding, like many others highlights both the importance of both the protein of interest and more generally cilia at large.

## 7. Conclusions

In conclusion, the primary cilium, once thought to be a vestigial process, has shown itself to be of increasing importance and complexity. Although a great deal of recent work has been directed to understanding the structure and function of this organelle, a great deal of work remains to be done before these structures and their components are completely understood. Notably, while mutational studies have allowed us to identify many components of the cilium and its associated pathways, more functional studies are required to dissect the meaningful protein interactions and their purpose in the broader context of cilia maintenance and cellular signaling. It is known that cilium serves as a structural scaffold for numerous specialized cell signaling pathways. More work is needed to truly understand how altering the physical properties of the cilia will affect the said pathways. Indeed, more fully understanding of the structure and regulation of cilia will likely result in not only a better understanding of the cilia and its associated pathways, a noble goal in and of itself, but also help us to progress with more tangible goals in terms of future medical treatment. Defects in ciliary biology have been implicated in a myriad of human disorders even beyond the well-known ciliopathies such as Polycystic Kidney, Bardet Beidl syndrome, and Gorlin Syndrome. Further understanding of the cilia is also likely to yield powerful tools in the struggle against cancer, other developmental disorders, and in the manipulation of stem cells. In short, this small organelle has big things in store for its future.
